# Comparison of the xylose reductase-xylitol dehydrogenase and the xylose isomerase pathways for xylose fermentation by recombinant *Saccharomyces cerevisiae*

**DOI:** 10.1186/1475-2859-6-5

**Published:** 2007-02-05

**Authors:** Kaisa Karhumaa, Rosa Garcia Sanchez, Bärbel Hahn-Hägerdal, Marie-F Gorwa-Grauslund

**Affiliations:** 1 Department of Applied Microbiology, Lund University, P.O.Box 124, SE-22100 Lund, Sweden

## Abstract

**Background:**

Two heterologous pathways have been used to construct recombinant xylose-fermenting *Saccharomyces cerevisiae *strains: i) the xylose reductase (XR) and xylitol dehydrogenase (XDH) pathway and ii) the xylose isomerase (XI) pathway. In the present study, the *Pichia stipitis *XR-XDH pathway and the *Piromyces *XI pathway were compared in an isogenic strain background, using a laboratory host strain with genetic modifications known to improve xylose fermentation (overexpressed xylulokinase, overexpressed non-oxidative pentose phosphate pathway and deletion of the aldose reductase gene *GRE3*). The two isogenic strains and the industrial xylose-fermenting strain TMB 3400 were studied regarding their xylose fermentation capacity in defined mineral medium and in undetoxified lignocellulosic hydrolysate.

**Results:**

In defined mineral medium, the xylose consumption rate, the specific ethanol productivity, and the final ethanol concentration were significantly higher in the XR- and XDH-carrying strain, whereas the highest ethanol yield was achieved with the strain carrying XI. While the laboratory strains only fermented a minor fraction of glucose in the undetoxified lignocellulose hydrolysate, the industrial strain TMB 3400 fermented nearly all the sugar available. Xylitol was formed by the XR-XDH-carrying strains only in mineral medium, whereas in lignocellulose hydrolysate no xylitol formation was detected.

**Conclusion:**

Despite by-product formation, the XR-XDH xylose utilization pathway resulted in faster ethanol production than using the best presently reported XI pathway in the strain background investigated. The need for robust industrial yeast strains for fermentation of undetoxified spruce hydrolysates was also confirmed.

## Background

**Figure 1:**
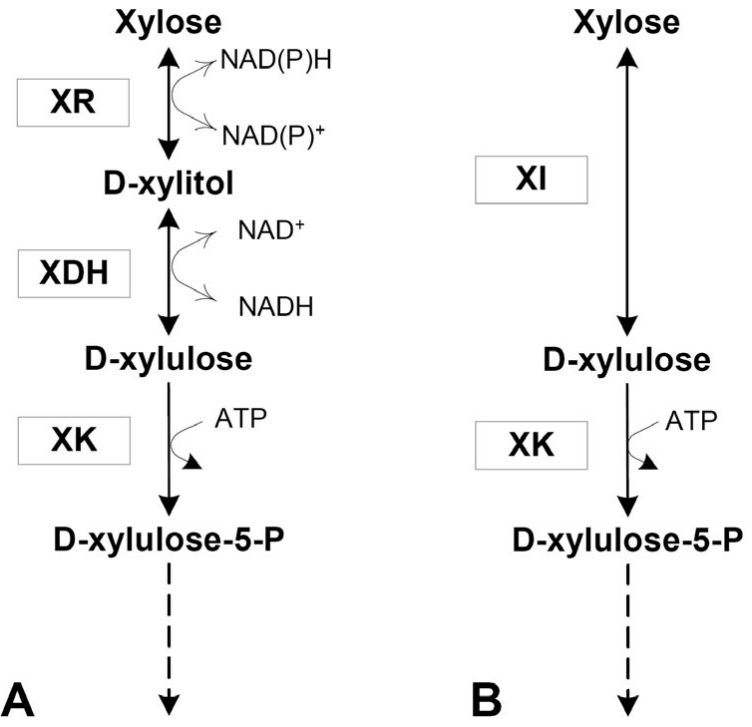
Metabolic pathways for xylose utilization. A. The XR-XDH pathway. B. The XI pathway.

*Saccharomyces cerevisiae *is a promising candidate for industrial bioethanol production due to its robustness, inhibitor tolerance and high ethanol productivity [1,2]. Efficient fermentation of pentose sugars is necessary to attain economically feasible processes for ethanol production from lignocellulosic biomass [3], however *S. cerevisiae *does not naturally ferment xylose. Anaerobic xylose fermentation by *S. cerevisiae *was first demonstrated by heterologous expression of xylose reductase (XR) and xylitol dehydrogenase (XDH) [4] from *Pichia stipitis *together with overexpression of the endogenous xylulokinase (XK) [5-8]. However, the difference in cofactor preference between the mainly NADPH-dependent XR and the strictly NAD^+^-dependent XDH may limit the flux from xylose to xylulose (Figure 1A), which has been supported by xylitol excretion observed in strains carrying XR and XDH [6-11]. Later, xylitol excretion has been reduced by metabolic engineering strategies such as optimisation of the expression levels of XR and XDH [12-15], changing the cofactor affinity of XR [16], or modifying the redox metabolism of the host cell [17-19].

To instead allow isomerization of xylose to xylulose (Figure 1B), heterologous expression of several xylose isomerases (XI) in *S. cerevisiae *has been attempted [20-26], of which the XI from the fungus *Piromyces *sp. [26] resulted in the highest activity. However, the sole expression of *Piromyces *XI did not result in appreciable growth on xylose [26]. Only when adaptation [27] or extensive genetic engineering [28] was also applied, were growth and fermentation on xylose achieved.

Genetic modifications other than the sole introduction of initial xylose utilization pathway are needed for efficient xylose metabolism. The combination of overexpressed XK, overexpressed non-oxidative pentose phosphate pathway (PPP) and deletion of the endogenous aldose reductase gene *GRE3 *have been shown to enhance both aerobic and anaerobic xylose utilization in XR-XDH- as well as XI- carrying strains [15,28,29]. The overexpression of XK is necessary to overcome the naturally low expression level of this enzyme [6,8,30,31], and the overexpression of the PPP enzymes enables efficient incorporation of xylulose-5-phosphate into the central metabolism [32]. Furthermore, the gene *GRE3 *codes for an unspecific reductase that functions as an NADPH-dependent xylose reductase [33], and contributes to xylitol formation [34,35] with concomitant inhibition of XI activity [36].

To the best of our knowledge, no comparative assessment has been reported for a pair of isogenic strains with the two functional xylose utilization pathways, XR and XDH from *P. stipitis*, or XI from *Piromyces *sp. In the present study, an engineered strain with overexpressed XK, overexpressed non-oxidative PPP and *GRE3 *deletion in CEN.PK background was used [29] to generate two isogenic strains that carried either the XR-XDH or the XI pathway. The xylose fermentation performance of the two strains was compared.

Recombinant xylose-fermenting strains are aimed to be used in industrial ethanol production from lignocellulose hydrolysates. The hydrolysates contain large amounts of inhibitory compounds such as furfural, hydroxymethyl furfural, acetate and phenolics, the concentrations of which depend on the raw material and hydrolysis method [37-39]. As industrial yeast strains are generally more tolerant to such inhibitors than laboratory strains [40,41], the two isogenic strains were also compared with the industrial xylose-fermenting strain TMB 3400 [42] with respect to hexose and pentose fermentation in an undetoxified lignocellulose hydrolysate.

## Results

### Strain construction

To generate an isogenic pair of strains, a strain with high-level expression of XI was constructed similar to the previously generated strain TMB 3057 with high-level expression of XR and XDH [29] (Table 1). The strain background TMB 3044, which contains overexpressed XK, overexpressed non-oxidative PPP and deletion of the aldose reductase gene *GRE3 *[29] (Table 1), was used. The *Piromyces *XI gene was cloned in the yeast multicopy plasmid YEplacHXT [29] between the truncated *HXT7 *promoter [43] and the *PGK1 *terminator. The truncated *HXT7 *promoter has been reported to give the highest expression levels compared with other yeast glycolytic promoters [43]. The plasmid containing the XI gene was then used to transform strain TMB 3044 [29], resulting in strain TMB 3066 (Table 1). Thus, strain TMB 3066, with high XI activity, and strain TMB 3057, with high XR and XDH activities, are isogenic with the exception of the plasmids containing the two xylose-utilization pathways.

**Table 1:** Strains and plasmids used in this study.

	*Relevant genotype*	*Xylose pathway*	*Reference*
Plasmids			
YEplacHXT	bla *HXT7p*-*PGK1t URA3*	-	[29]
YEpHXT-XIp	bla *HXT7p*-XI (*Piromyces*)-*PGK1t URA3*	XI	This study
*S. cerevisiae*			
TMB 3044	CEN.PK 2-1C, *Δgre3, his3::PGK1p-XKS1-PGK1t, TAL1::PGK1p-TAL1-PGK1t, TKL1::PGK1p-TKL1-PGK1t, RKI1::PGK1p-RKI1-PGK1t, RPE1::PGK1p-RPE1-PGK1t*,	-	[29]
TMB 3057	TMB 3044, pY7 (*ADH1p-XYL1-ADH2t, PGK1p-XYL2-PGK1t URA3*)	XR-XDH-XK	[29]
TMB 3066	TMB 3044, YEpHXT-XIp (*HXT7p*-XI-*PGK1t URA3*)	XI-XK	This study
TMB 3400	*HIS3::(ADH1p-XYL1-ADH2t, PGK1p-XYL2-PGK1t, PGK1p-XKS1-PGK1t*)	XR-XDH-XK	[42]

**Figure 2:**
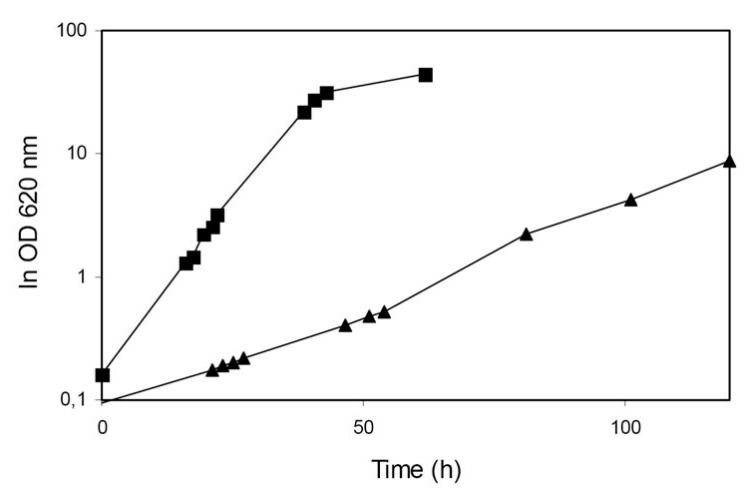
Aerobic growth of TMB 3057 (XR-XDH) (■) and TMB 3066 (XI) (▲) in mineral medium with xylose (50 g/l) as the sole carbon source.

Strain TMB 3066 with XI grew aerobically on xylose at a growth rate of 0.02 h^-1^, whereas a growth rate of 0.16 h^-1 ^has previously been obtained for strain TMB 3057 with XR and XDH under same conditions [29] (Figure 2). Anaerobic growth was not measured. XI activity in strain TMB 3066 was 0.82 ± 0.01 U/mg protein.

### Anaerobic xylose fermentation in mineral medium

**Figure 3:**
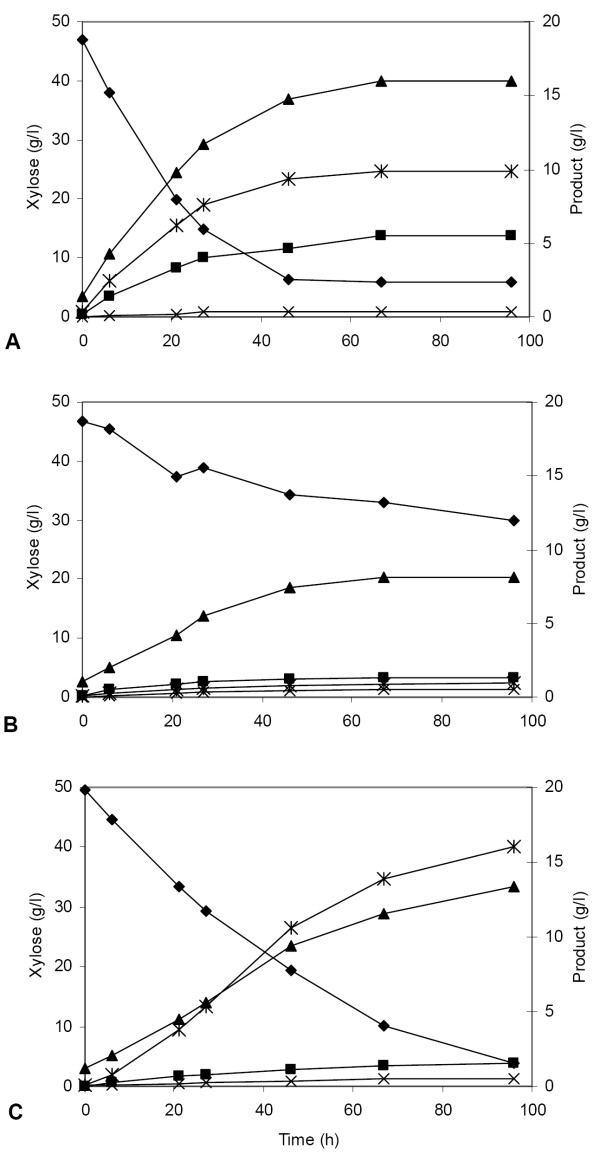
Anaerobic fermentation profiles of strains TMB 3057 (*P. stipitis *XR and XDH on plasmid) (A), TMB 3066 (*Piromyces *XI on plasmid) (B) and TMB 3400 (industrial strain with chromosomally integrated *P. stipitis *XR and XDH) (C). Mineral medium with xylose (50 g/l) was used. The initial biomass concentration for all strains was 5 g/l. Symbols: ◆ xylose; * xylitol; ■ glycerol; ▲ethanol; × acetate.

Xylose fermentation by strains TMB 3057 and TMB 3066 was compared in anaerobic batch culture. The industrial strain TMB 3400 with chromosomally integrated XR-XDH genes [42] was also included in the comparison. Buffered mineral medium containing xylose (50 g/l) and an initial biomass concentration of 5 g dry weight/l were used. Representative fermentation profiles for the three strains are shown in Figure 3.

The XR-XDH-carrying strain TMB 3057 consumed xylose at a rate of 0.13 g xylose g cells^-1 ^h^-1 ^(Table 2), with ethanol, xylitol and glycerol as fermentation products (Figure 3A). The isogenic strain TMB 3066 carrying XI consumed xylose at a 2.5 fold lower rate of 0.05 g xylose g cells^-1 ^h^-1 ^(Table 2). Ethanol was the major fermentation product of TMB 3066 (Figure 3B), with a yield of 0.43 g g consumed xylose^-1^, which is close to the theoretical yield of 0.51 g g consumed xylose^-1 ^(Table 2). Despite the formation of the by-products, TMB 3057 produced twice as much ethanol (13.3 g/l) within 50 hours as a result of the higher xylose consumption rate. The specific ethanol productivity was 2-fold higher in the XR-XDH-carrying strain TMB 3057 than in the XI-carrying strain TMB 3066 (Table 2). The industrial strain TMB 3400 consumed xylose more slowly than the laboratory strain TMB 3057 (Table 2) (Figure 3C).

**Table 2:** Results of anaerobic batch fermentation of xylose (50 g/l) by strains TMB 3057, TMB 3066 and TMB 3400.

						**Yield**		
						
**Strain**	**Relevant genotype**	**Xylose consumed in 100 h**	**Final ethanol concentration**	**Specific xylose consumption rate**	**Specific ethanol productivity**	**Ethanol**	**Xylitol**	**Glycerol**
		g l^-1^	g l^-1^	g xylose g cells^-1 ^h^-1^	g ethanol g cells^-1 ^h^-1^	g g consumed xylose^-1^	g g consumed xylose^-1^	g g consumed xylose^-1^

TMB 3057	XR-XDH, plasmid	39.6 ± 3.4	13.3 ± 1.7	0.13 ± 0.04	0.04 ± 0.01	0.33 ± 0.02	0.22 ± 0.03	0.11 ± 0.02
TMB 3066	XI, plasmid	16.8 ± 3.8	7.3 ± 2.1	0.05 ± 0.02	0.02 ± 0.01	0.43 ± 0.03	0.04 ± 0.02	0.07 ± 0.02
TMB 3400	XR-XDH, integrated	36.5 ± 10.8	12.1 ± 2.5	0.06 ± 0.02	0.02 ± 0.01	0.34 ± 0.03	0.29 ± 0.07	0.04 ± 0.01

### Fermentation of lignocellulose hydrolysate

**Figure 4:**
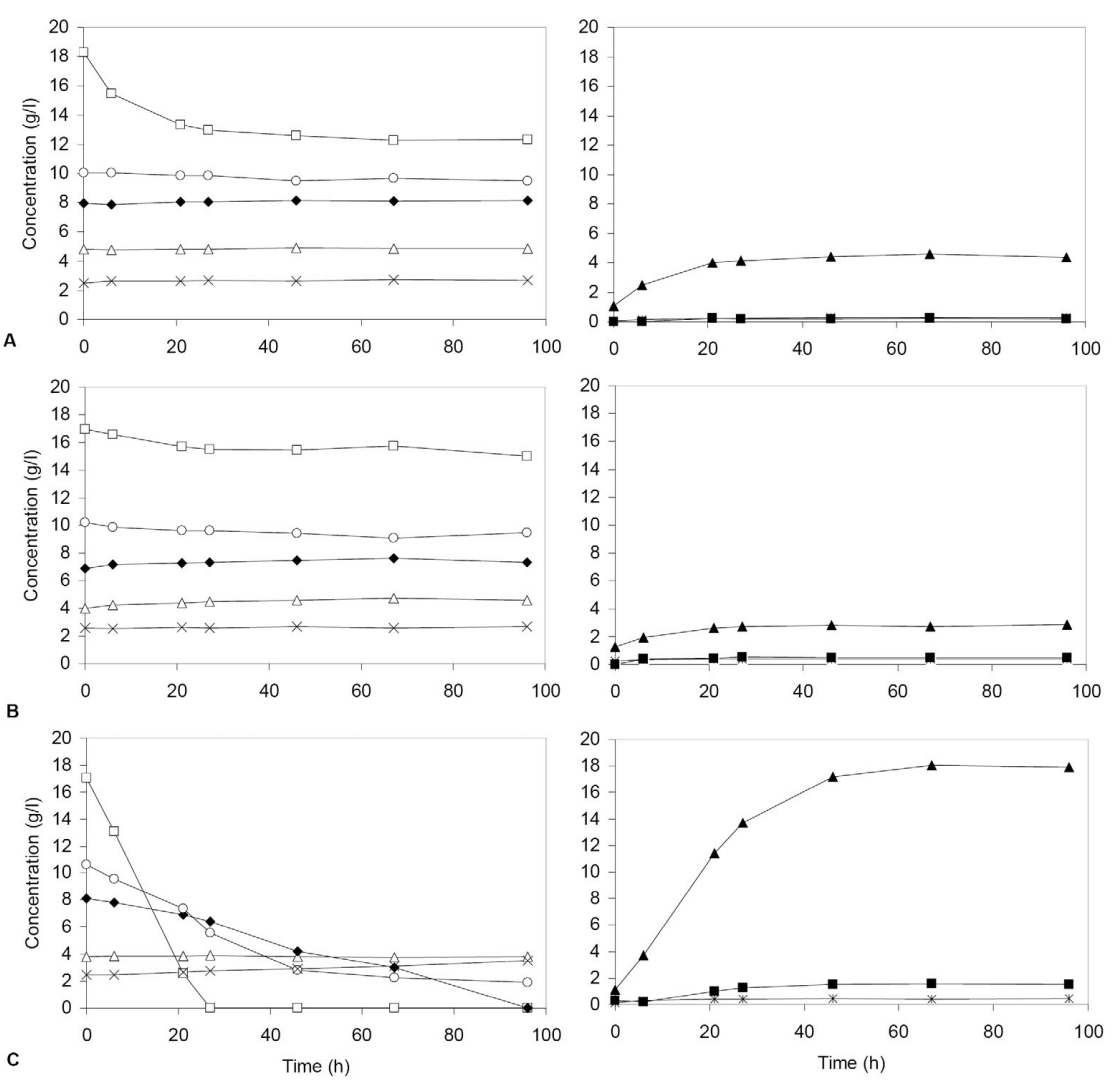
Fermentation of lignocellulose hydrolysate by strains TMB 3057 (*P. stipitis *XR and XDH on plasmid) (A), TMB 3066 (*Piromyces *XI on plasmid) (B) and TMB 3400 (industrial strain with chromosomally integrated XR and XDH) (C). The initial cell concentration was 5–10 g/l for all the fermentation experiments shown. For clarity, the hydrolysate components and their consumption are shown on the left, and the accumulation of the products is shown on the right. Symbols: ○ mannose; □glucose; Δ galactose; ◆ xylose; *xylitol; ■ glycerol; ▲ethanol; × acetate.

The three strains studied were also compared in batch fermentation of an undetoxified spruce hydrolysate [44]. The initial concentrations of the hydrolysate components were as follows: glucose, 16 g/l; mannose, 10 g/l; galactose, 4 g/l; xylose, 7 g/l; arabinose, 3 g/l; and acetic acid, 2.5 g/l. Fermentation was started with an initial biomass concentration of 5–10 g dry weight/l and pH 5.5. Representative fermentation profiles are shown in Figure 4, and the fermentation parameters are summarized in Table 3.

**Table 3:** Results of anaerobic batch fermentation of undetoxified lignocellulose hydrolysate by strains TMB 3057, TMB 3066 and TMB 3400.

						**Yield**		
						
**Strain**	**Relevant genotype**	**Glucose consumption rate**	**Mannose consumption rate**	**Xylose consumption rate**	**Specific ethanol productivity**	**Ethanol**	**Glycerol**	**Xylitol**
		g glucose g cells^-1 ^h^-1^	g mannose g cells^-1 ^h^-1^	g xylose g cells^-1 ^h^-1^	g ethanol g cells^-1 ^h^-1^	g g total sugar ^-1^	g g total sugar^-1^	g g xylose^-1^

TMB 3057	XR-XDH, laboratory strain	0.008 ± 0.003	0	0	0.005 ± 0.002	0.08 ± 0.02	0.006 ± 0.001	0
TMB 3066	XI, laboratory strain	0	0	0	0	0	0	0
TMB 3400	XR-XDH, industrial strain	0.021 ± 0.013	0.013 ± 0.01	0.005 ± 0.001	0.020 ± 0.012	0.41 ± 0.02	0.035 ± 0.006	0

Only a small amount of glucose was consumed by the two laboratory strains (Figures 4A, 4B), whereas nearly all glucose, mannose and xylose were consumed by the industrial strain TMB 3400 (Figure 4C). The final ethanol concentration was about 3 g/l for TMB 3057 (XR-XDH), 2 g/l for TMB 3066 (XI), and 16 g/l for the strain TMB 3400 (the XR-XDH-carrying industrial strain) (Figure 4). No xylitol was produced by TMB 3400, although nearly all the xylose was consumed within 100 h (Figure 4C).

## Discussion

Isogenic strains were used to compare the efficiency of xylose fermentation via the *P. stipitis *XR and XDH route and the *Piromyces *XI route. Comparing the two pathways in isogenic strains eliminates the influence of strain background on xylose uptake rate and product formation. In anaerobic batch fermentation of xylose in mineral medium, the xylose consumption rate was higher in the XR-XDH-carrying strain TMB 3057 than in the XI-carrying TMB 3066 in the strain background used. However, the XI-carrying strain TMB 3066 produced ethanol at almost theoretical yield. Nevertheless, TMB 3057 produced twice as much ethanol due to faster and almost complete xylose consumption. This is in accordance with the 8-fold higher aerobic growth rate that was observed on xylose for the XR-XDH-carrying strain (Figure 2).

The XI-carrying strain TMB 3066 reported in the present study displayed slower aerobic growth on xylose and slower anaerobic xylose fermentation than the previously designed strain RWB217 [28] (Table 4) with identical reported genetic modifications. The aerobic growth rate of TMB 3066 on xylose was 0.02 h^-1^, compared to 0.22 h^-1 ^measured for strain RWB217 in the previous investigation [28]. The slower xylose utilization of TMB 3066 cannot be attributed to a lower XI level, since the XI activity (0.82 U mg protein^-1^) was in the same range as that of the previously reported strain (0.3–1.1 U mg protein^-1^) [26] (Table 4). Thus, the reason for the slower growth and fermentation of the XI-carrying strain TMB 3066 in the present study probably lies in the strain background. Both strains originate from a CEN.PK laboratory strain and nearly identical genetic modifications were made [29]. This suggests that the xylose fermentation capacity of the RWB217 must be ascribed to unknown strain characteristics. For example, this may result from higher activity levels of XK or the PPP enzymes in RWB217 than in TMB 3057, that could have originated from mutagenic events during the transformation process. While the activities of XK and the PPP enzymes have been reported for TMB 3057 [29], this information is not available for RWB217 [28] (Table 4).

**Table 4:** Comparison of XI and XR-XDH-carrying laboratory strains and the industrial XR-XDH carrying strain TMB 3400.

		**Anaerobic fermentation**		**Enzyme activity U/mg protein**								**Reference**
		
**Strain**	**Aerobic growth rate xylose **h^-1^	**Xylose cons. rate **g xylose g cells^-1 ^h^-1^	**Ethanol yield **g ethanol g consumed sugar^-1^	**XI**	**XR**	**XDH**	**XK**	**TAL**	**TKL**	**RKI**	**RPE**	
TMB 3057	0.16	0.13	0.33	-	0.35	2.16	0.89*	0.91*	0.06*	0.14*	0.09*	[29]
TMB 3066	0.02	0.05	0.43	0.82	-	-	0.89*	0.91*	0.06*	0.14*	0.09*	This work
RWB217	0.22	0.50 **	0.43	0.3–1.1	-	-	+	+	+	+	+	[28]
TMB 3400	0.14	0.06	0.34	-	0.09	0.59	+	↑	↑	n.a.	n.a.	[55]

Aerobic growth rates on xylose, average xylose consumption rate for 50 hours of fermentation, and ethanol yield for anaerobic xylose fermentation are presented together with the enzyme activities of relevant enzymes are presented. (+ overexpressed by genetic engineering; ↑ upregulated upon mutagenesis).

* Measured for strain background TMB 3044

** Average rate for batch fermentation recalculated from [28] to allow comparison

The comparison of xylose fermentation with the isogenic strains demonstrates that the *Piromyces *XI activity is not sufficient to ensure the same growth and fermentation rates as those obtained with the XR-XDH pathway in the strain background investigated. Highest possible XI activity among the presently known XI genes was used, since the *Piromyces *XI gene was expressed on a multicopy plasmid from the strongest known yeast promoter [43]. When the XI gene was chromosomally integrated in the same strain background that was used for constructing TMB 3057 and TMB 3066, aerobic growth on xylose was not achieved (data not shown). However, chromosomal integration is a prerequisite to render industrial production organisms genetic stability [45], and this can not be obtained with multicopy plasmid-carrying strains [46,47]. As opposed to XI, chromosomal integration of XR and XDH in industrial strain background is sufficient to enable xylose fermentation in lignocellulose hydrolysates, as demonstrated for strain TMB 3400 [48,49] (Figure 4).

The difference in xylose consumption between the XR-XDH- and XI-carrying isogenic strains may be partly explained by the kinetic properties of the enzymes involved. The K_m _of the *Piromyces *XI for xylose has been estimated to about 20 mM [26], while the K_m _of the *P. stipitis *XR for xylose ranges from 42 mM [50] to 97 mM [51]. No other kinetic information is available for the *Piromyces *XI. The specific activity of purified bacterial XIs ranges from 2 to 20 U mg protein^-1 ^[52], while for *P. stipitis *XR it is 17–48 U mg protein^-1 ^[50,51]. Although the *Piromyces *XI has a slightly higher affinity for xylose, the higher specific activity of XR may result in more efficient *in vivo *conversion of xylose to xylitol, and thereafter to xylulose. A XI with higher specific activity and improved kinetic properties could be generated by mutagenesis [53] or gene shuffling [54], in combination with an efficient screening strain [29] to allow faster xylose utilization and chromosomal integration.

The industrial strain TMB 3400 with chromosomally integrated XR and XDH genes [42] was included in the present comparison because it ferments xylose in both mineral medium [41] and in undetoxified lignocellulose hydrolysate [48]. In mineral medium TMB 3400 consumed xylose more slowly and produced more xylitol than TMB 3057, most likely due to the lower XR and XDH levels [15,55] resulting from chromosomal integration of the XR and XDH genes, compared with plasmid-based expression in TMB 3057.

Because the design of xylose-fermenting strains for bioethanol production aims at fermentation of industrial substrates, the three strains TMB 3057 (XR-XDH), TMB 3066 (XI) and TMB 3400 (integrated XR-XDH) were also compared regarding the fermentation of an undetoxified spruce hydrolysate. Laboratory strains are considered to be less tolerant than industrial strains to inhibitory compounds present in lignocellulose hydrolysates [40,41,56]. For instance, the industrial strains TMB 3400 and TMB 3000 were shown to retain viability and fermentation capacity in lignocellulosic hydrolysates whereas the laboratory strain CBS 8066 did not [48,57]. This was confirmed here by the observation that the CEN.PK based laboratory strains of this study did not significantly ferment the investigated undetoxified spruce hydrolysate, whereas the industrial strain TMB 3400 produced ethanol from most of the sugar present. A slightly higher amount of glucose was consumed by the XR-XDH-carrying laboratory strain TMB 3057 than by the XI-carrying laboratory strain TMB 3066, which may reflect the possible function of the heterologous XR in stress response, similarly to the native aldose reductases in *S. cerevisiae *[35,58].

Nearly all the xylose was consumed by TMB 3400 in the hydrolysate, with ethanol as the main product. No xylitol was produced. The absence of xylitol production in undetoxified lignocellulose hydrolysate is in agreement with previous studies, in which the presence of external electron acceptors such as furfural reduced xylitol formation [59-61]. Absence of xylitol formation has frequently been observed in lignocellulose fermentation with XR- and XDH-carrying strains [48,49,62-64]. Indeed, in a comparison of different hydrolysates for fermentability by an XR-XDH-carrying strain, xylitol formation was only detected in a corn fiber hydrolysate [49], which is known to contain less furfural than softwood hydrolysates [65].

## Conclusion

When comparing two isogenic strains with high-level expression of the XR-XDH pathway or the XI pathway, higher specific ethanol productivity was observed with the XR-XDH pathway as a result of significantly higher xylose consumption rate in the strain background investigated. On the other hand, the ethanol yield was higher in the XI carrying strain. The CEN.PK-based laboratory strains did not ferment the lignocellulose hydrolysate used in this study, demonstrating that robust industrial strains are essential for the fermentation of industrial substrates. Xylitol formation, which has been often described as being the major drawback of the XR-XDH strategy, was not observed in the fermentation of lignocellulosic hydrolysate by TMB 3400.

## Methods

### Strains and media

Plasmids and yeast strains used in this study are summarized in Table 1. *Escherichia coli *strain DH5α (Life Technologies, Rockville, MD, USA) was used for cloning. *E. coli *was grown in LB medium [66] supplemented with ampicillin (100 mg/l). Liquid cultures of *S. cerevisiae *were grown in yeast nitrogen base medium (YNB) (Difco YNB without amino acids (6.7 g/l), Becton, Dickinson and Company, Sparks, MD, USA) supplemented with glucose (20 g/l) or xylose (50 g/l) (Acros Organics, NJ, USA) and buffered at pH 5.5 with potassium hydrogen pththalate (10.2 g/l) [37]. YPD or YNB plates were used for plate cultures. When required, the medium was supplemented with uracil (40 μg/ml).

Lignocellulose hydrolysate generated by two-step dilute acid hydrolysis of spruce [44] was used. The hydrolysate was stored at 4°C at pH 1.5. Before usage, the pH was adjusted to 5.5 and the hydrolysate was sterile filtered.

### Molecular biology methods and cloning

Standard molecular biology techniques were used [66]. The calcium chloride method was used for transformation of *E. coli *[67] and the lithium acetate method was used for transformation of *S. cerevisiae *[68]. Taq polymerase, restriction enzymes and T4-nucleotide ligase were purchased from Fermentas (Vilnius, Lithuania). DNA sequencing was performed with an Abi-Prism BigDye cycle sequencing kit (Applied Biosystems, Weiterstadt, Germany).

The gene coding for the *Piromyces *sp. XI was commercially synthesized and cloned in plasmid YEplacHXT with the restriction enzymes *Bam*HI and *Pst*I. Correct ligation and gene sequence was verified by electrophoresis, PCR and sequencing. The resultant multicopy plasmid YEpHXT-XIp was used to transform *S. cerevisiae *TMB 3044 [29] and transformants were selected for prototrophy on YNB plates containing glucose. The resultant strain was named TMB 3066.

### XI activity

Cells were grown in YNB medium containing xylose and harvested in mid-exponential growth phase. Cells were washed with sterile water and proteins were extracted with yeast protein extract solution Y-PER (Pierce, Rockford, IL, USA) according to the manufacturer's instructions. Protein concentrations were determined with Coomassie protein assay reagent (Pierce) according to the manufacturer's instructions. The XI activity was determined using sorbitol dehydrogenase as previously described [26] at 30°C. A U-2000 spectrophotometer (Hitachi, Tokyo, Japan) was used for the measurements, which were performed for biological triplicate samples.

### Growth experiments

Pre-cultures were cultivated until the late exponential phase in YNB medium with glucose. Cells were inoculated at an initial OD_620nm _of 0.2 in YNB medium with xylose (50 g/l), and grown in shake flasks at 30°C with 200 rpm agitation (INR-200 shake incubator, Gallenkamp, Leicester, UK). Experiments were repeated at least twice.

### Batch fermentation

Pre-cultures were grown until the late exponential growth phase (OD_620nm_20) in YNB medium with xylose (50 g/l). Cells were harvested by centrifugation for 3 minutes at 4°C, after which the cell pellets were mixed with the fermentation medium. Anaerobic batch fermentation was performed at 30°C in 25 ml serum flasks containing 23 ml medium. The flasks were sealed with rubber stoppers into which cannulas were inserted for sampling and the release of exhaust gas, and were stirred with small magnetic stirrers at 150 rpm. A layer of mineral oil was added to ensure anaerobiosis. Samples were withdrawn during 100 hours of fermentation. Experiments were performed at least in duplicate.

For fermentation in mineral medium, YNB medium containing xylose (50 g/l) was used, complemented with potassium hydrogen phthalate buffer (pH 5.5) [37], Tween 80 (0.4 g/l) and ergosterol (0.01 g/l). Lignocellulose hydrolysate was complemented with concentrated YNB, Tween 80 (0.4 g/l) and ergosterol (0.01 g/l). The total volume of the added medium components did not exceed 10% of the culture volume. Tween 80 and ergosterol were added dissolved in ethanol, which resulted in an initial ethanol concentration of about 1 g/l, which was taken into account in all calculations. The initial cell concentration was 5 g dry weight/l for the xylose fermentation and 5–10 g dry weight/l for lignocellulose hydrolysate fermentation.

### Analyses

Concentrations of xylose, xylitol, glycerol, acetic acid and ethanol from fermentation in mineral medium were analysed by HPLC (Waters, Milford, Massachusetts, USA) with an Aminex HPX-87H ion-exchange column (Bio-Rad, Hercules, USA). A mobile phase of 5 mM H_2_SO_4 _was used at a flow rate 0.6 ml/min and the column temperature was 45°C. For xylose fermentation experiments, the ethanol concentration was corrected for evaporation with the help of degree of reduction balance calculation. In the case of hydrolysate fermentation, the concentrations of glucose, xylose, galactose, arabinose, glycerol, xylitol and arabitol were analysed with HPLC (Waters, Milford, Massachusetts, USA) using an HPX-87P (Bio-Rad, Hercules, USA) ion exchange column at 85°C with water as the mobile phase at a flow rate of 0.5 ml/min. Glucose, galactose, mannose, glycerol, acetic acid and ethanol were analysed by HPLC (Beckman Instruments, Fullerton, CA, USA) using three Aminex HPX-87H ion exchange columns (Bio-Rad, Hercules, USA) connected in series, to allow separation of the peaks. A temperature of 45°C and a mobile phase of 5 mM H_2_SO_4 _at a flow rate 0.6 ml/min were used.

Cell dry weight was determined in triplicate by filtering 1 ml of the culture with a pre-weighed nitrocellulose filter with 0.45 μm pores. Filters were dried in a microwave oven and weighed.

## Authors' contributions

KK participated in the design of the study, performed cloning, strain construction and fermentation experiments, analyzed the data and wrote the manuscript.

RGS participated in design and performance of cloning and strain construction.

BHH participated in design of the study and commented the manuscript.

MFGG participated in design of the study and commented the manuscript.

All authors have read and approved the manuscript.
